# Supplementary material for: Tools for Genetic Engineering and Gene Expression Control in Novosphingobium aromaticivorans and Rhodobacter sphaeroides
Source: bioRxiv. 2023 Aug 26:2023.08.25.554875. Preprint. [Version 1] doi: 10.1101/2023.08.25.554875 (PMC10473679; doi:10.1101/2023.08.25.554875)

bioRxiv preprint doi: https://doi.org/10.1101/2023.08.25.554875; this version posted August 26, 2023. The copyright holder for this preprint (which was not certified by peer review) is the author/funder, who has granted bioRxiv a license to display the preprint in perpetuity. It is made available under aCC-BY 4.0 International license.

Figure S1: Confirmation of att::Tn*7* integration. A: Schematic of *glmS* genetic region with and without att::Tn7 integration and locations of validation primers. B: *N. aromaticivorans* clones. C: *R. sphaeroides* clones.

Figure S2: CRISPRi knockdown of *mScarlet-I* with initial Mobile-CRISPRi constructs. Constructs with both sgRNA and dCas9 under the control of PlLacO1 were tested with three different targeting sgRNAs in *E. coli* (A), *N. aromaticivorans* (B), and *R. sphaeroides* (C). NTC=Non-targeting control strain.

Figure S3: Expanded data for *murC* CRISPRi in *N. aromaticivorans* and *R. sphaeroides*. A: 10-fold serial dilutions of *N. aromaticivorans* with MCi constructs with a sgRNA targeting the essential gene *murC* or a non-targeting control sgRNA. *N. aromaticivorans* cells were normalized to an OD600 of 10 prior to serial dilution. Cells were grown on rich media (464a) in the presence or absence of 1mM IPTG. B: 10-fold serial dilutions of *R. sphaeroides* with MCi constructs with a sgRNA targeting the essential gene *murC* or a non-targeting control sgRNA. *R. sphaeroides* cells were normalized to an OD600 of 10 prior to serial dilution. Cells were grown on rich media (LB) in the presence or absence of 1mM IPTG. Samples presented in the main text are indicated in gray.

bioRxiv preprint doi: https://doi.org/10.1101/2023.08.25.554875; this version posted August 26, 2023. The copyright holder for this preprint (which was not certified by peer review) is the author/funder, who has granted bioRxiv a license to display the preprint in perpetuity. It is made available under aCC-BY 4.0 International license.

Figure S1

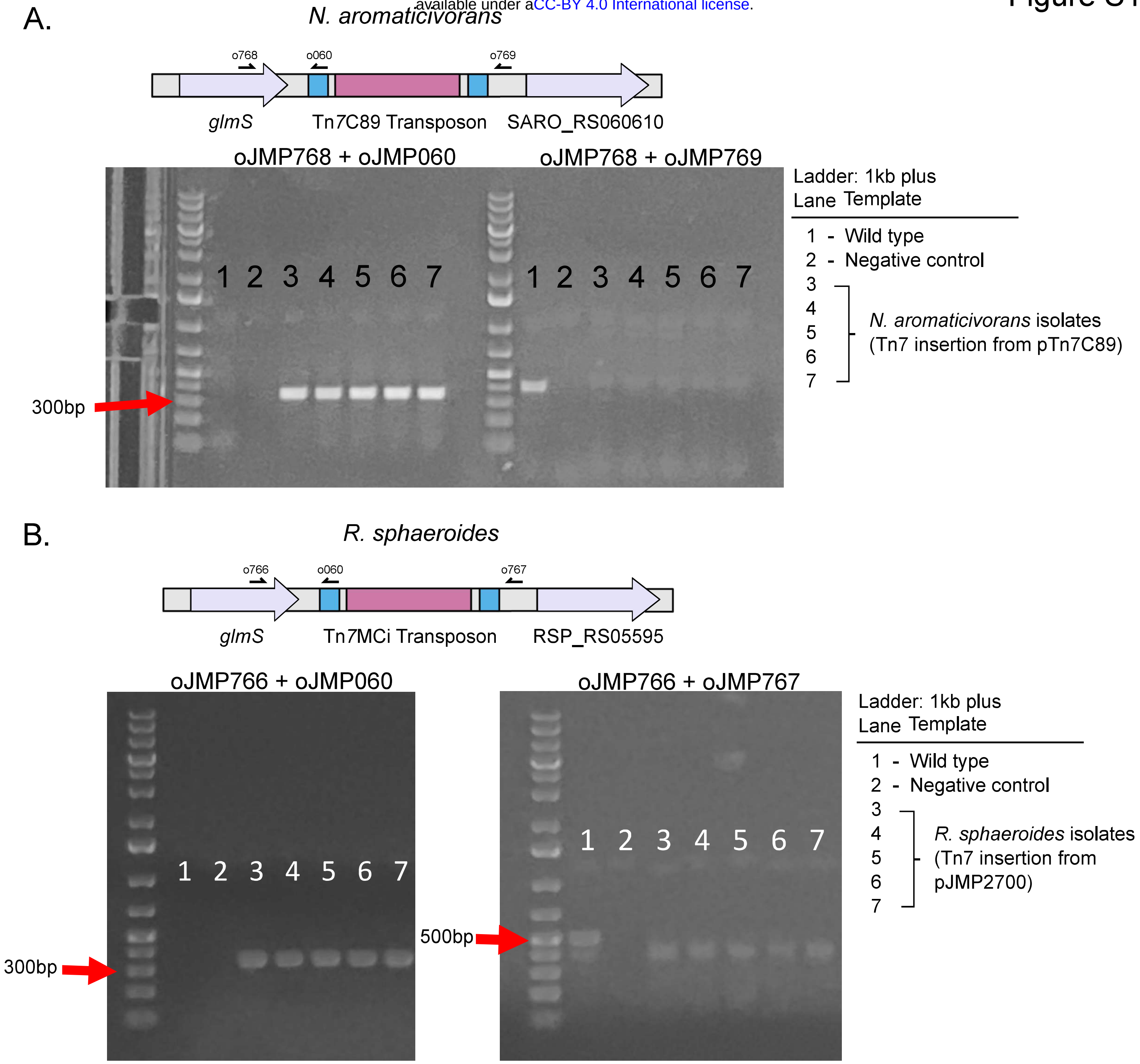

bioRxiv preprint doi: https://doi.org/10.1101/2023.08.25.554875; this version posted August 26, 2023. The copyright holder for this preprint (which was not certified by peer review) is the author/funder, who has granted bioRxiv a license to display the preprint in perpetuity. It is made available under aCC-BY 4.0 International license.

Figure S2

A.

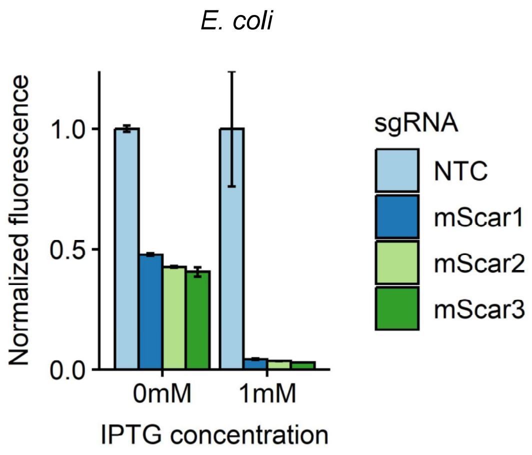

B.

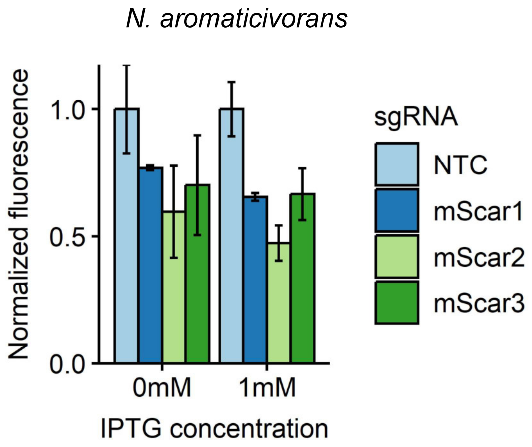

C.

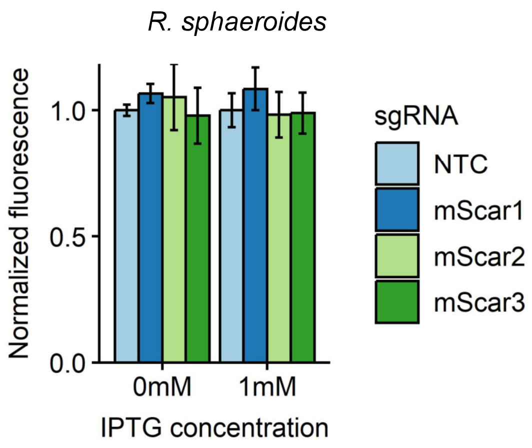

bioRxiv preprint doi: https://doi.org/10.1101/2023.08.25.554875; this version posted August 26, 2023. The copyright holder for this preprint (which was not certified by peer review) is the author/funder, who has granted bioRxiv a license to display the preprint in perpetuity. It is made available under aCC-BY 4.0 International license.

Figure S3

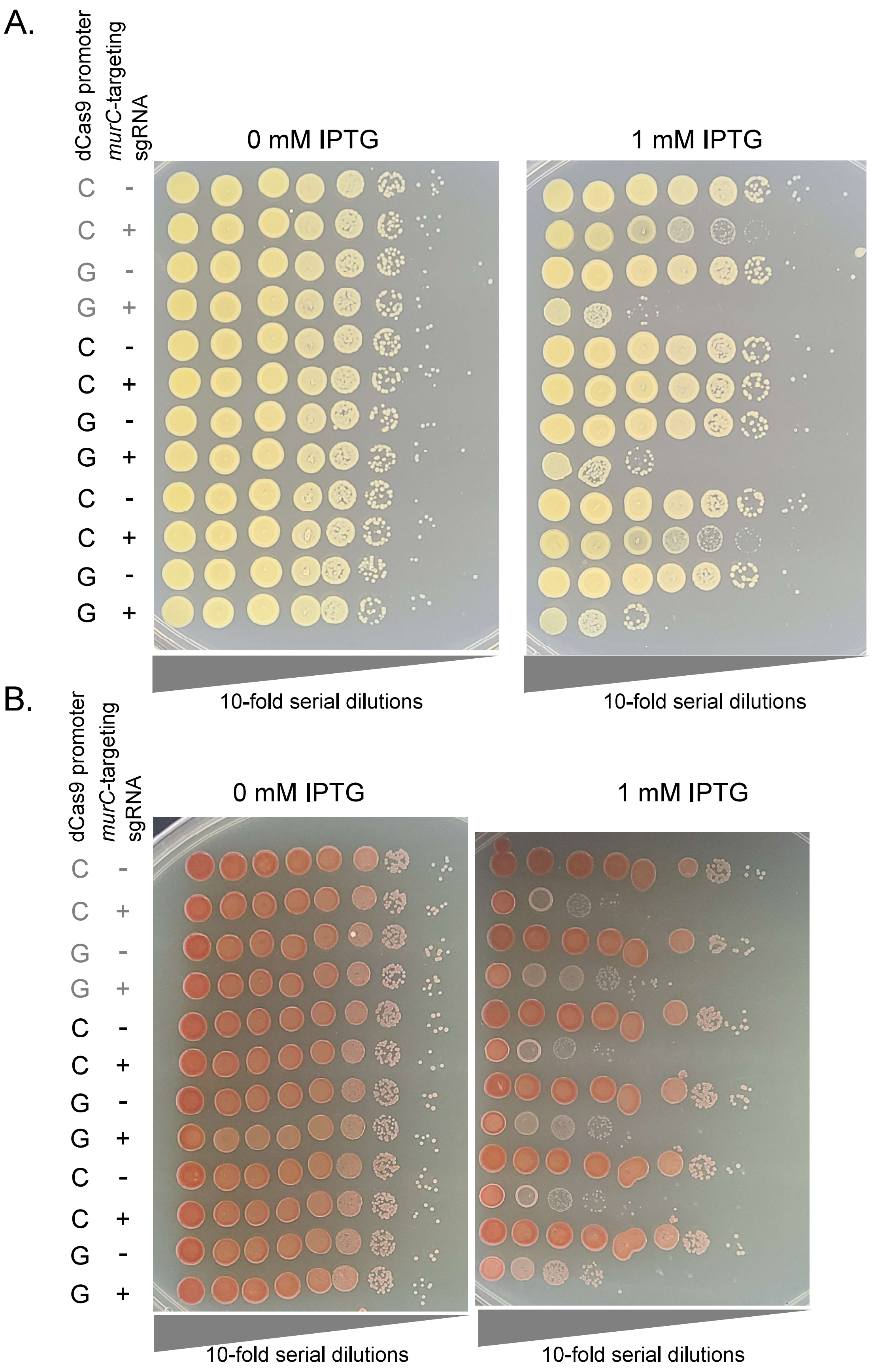

Supplement: Supplement 1 [file NIHPP2023.08.25.554875v1-supplement-1.pdf]
